# To Stop Coughing

**Published:** 1882-03

**Authors:** 


### TO STOP COUGHING.

If there is one truth more than another persistently taught in these pages, it is that it is generally dangerous and always injurious to "stop a cough." When a man has consumption, and his cough is stopped by anything he swallows, or if it suddenly stops itself, he will die in a week, because cough is nature's means of bringing the phlegm up from the lungs; all consumptives will testify that the more freely they can "bring up," the better they feel, simply because what is brought up comes from the lungs, leaves more room for air, they breathe freer and fuller. The cough brings the matter from the lungs to the top of the throat; from that point it is brought with a hack or a hem, into the mouth, from which it is passed out by the act of expectoration; if there was no cough, there would be an inevitable accumulation in the lungs, until they would be filled with yellow matter; no air could penetrate, and death would necessarily ensue. Merely "stopping" a cough, which is the effect aimed at by all medicines sold for coughs, colds and consumptions, not only does nothing towards effecting a cure, but counteracts nature in her efforts to do the same, hence tend to destroy, instead of preserve: of all the medicines known, and which are given in reference to coughs, opium is the most pernicious ultimately; whether it be in the shape of the crude material itself, or paregoric, or laudnam or morphine; because, if it alone is relied upon, it is like preventing the appearance of smoke on board ship, while the hold remains on fire, and is every moment in process of destruction. When these medicines are ignorantly given to children for cough, or pain, or bowel complaint, they have an immediate but deceptive good effect, to be followed with convulsions or water on the brain; this accounts for the thousands of deaths of young children in summer time by fits and convulsions.

Let it be remembered by our readers that we have steadily aimed to avoid giving medical prescriptions in the pages of this Journal, only making an exception in case of cholera, and then merely for the purpose of arresting the progress of the disease, until a physician could be secured. When a person is sick and actually needs medicine, he should send for a physician, he ought no more to medicate himself than to mend his watch, or repair an old shoe. Physicians themselves after half a century's experience, sometimes mistake the meanings of a symptom, and a mistake in certain cases, is death. The constant aim of this Journal is two-fold. First, to show how to avoid sickness; second, to teach what may be done towards restoration by prompt attention, good nursing and the use of diet, exercise and air; but if more is needed, by all means send for a resident physician.

**THERMOMETERS.**—Most persons know that sudden changes of weather endanger the health, and not unfrequently occasion the loss of valuable lives. These sudden and great changes often take place during the night. But it requires a day or two for the cold to get into the house, with the result that a person gets up, dresses, and gets into the street, before he discovers that it is fifteen, twenty, or thirty degrees (or even more) colder than it was the day previous; but before he has arrived at the knowledge of this fact, he has been chilled, or has started on a journey, or has got so far from home that a change of clothing is very inconvenient, if not almost impracticable. Franklin, Abbott Lawrence, Rachel the tragedienne, and other eminent personages, lost their lives by dressing for a temperature, or exposing themselves to a degree of coldness in the atmosphere, of which they were not conscious.

It is said of the Duke of Wellington, that he had every variety of garment; that his servant dare not bring any one of them to him, until he had gone himself to the window, hoisted it, and by the protrusion of his immense long nose out into the atmosphere, had calculated with this new feeler, the state of the weather, and the coat adapted to it.

At all events, the Duke lived a long time, and the habit referred to was a wise one, by whomsoever practiced. The lesson is this: Judicious attention to the state of the thermometer, at least every morning, would answer a wise purpose, by acquainting us with the state of the weather out of doors, thus enabling us to dress in reference to it. Every family who can possibly afford it, should have a thermometer, of easy access in the lower hall, in each sitting room, and a large one, with a red column, at the most northern exposure practicable, outside, in a situation easily accessible to every member of the family, without raising the window or opening the door

